# Deciphering the Role of Bovine Viral Diarrhea Virus Non-Structural NS4B Protein in Viral Pathogenesis

**DOI:** 10.3390/vetsci7040169

**Published:** 2020-10-31

**Authors:** Shahbaz Bashir, Andrey Kossarev, Violeta Cascon Martin, Jan Paeshuyse

**Affiliations:** Laboratory of Host Pathogen Interaction in Livestock, Department of Biosystems, Division of Animal and Human Health Engineering, KU Leuven University, 3000 Leuven, Belgium; shahbaz.bashir@kuleuven.be (S.B.); andrey.kossarev@kuleuven.be (A.K.); violeta.cascon@hotmail.com (V.C.M.)

**Keywords:** bovine viral diarrhea virus, NS4B, immunogenic, antibodies, western blot

## Abstract

Bovine viral diarrhea virus (BVDV) is a (+) ssRNA virus that belongs to the family *Flaviviridae*. BVDV is a significant animal pathogen causing substantial economic losses to the cattle industry worldwide through respiratory and gastrointestinal infections and abortion or birth of persistently infected calves. While the immunogenic profile of some of the BVDV proteins (i.e., E^rns^, E2 and NS3) is well established during viral pathogenesis, very little information is available about most of BVDV’s non-structural proteins in this regard. In recent times, the NS4B protein has emerged as an interesting target of diagnostic, vaccination and therapeutic value in viral infections of other members of the family *Flaviviridae* due to its key scaffold-like contribution in the viral replication complex. Although, BVDV-NS4B has a membrane topology alongside its role in induction of autophagosomes *in vitro*. However, information on its immunogenicity during BVDV pathogenesis and vaccination is scarce. To characterize the immunogenic profile of the NS4B, five cows were vaccinated with the live attenuated BVDV vaccine Bovela^®^ and blood samples were taken pre- and post-immunization for serum isolation. Virus neutralization assay (VNA) confirmed the presence of anti-BVDV antibodies in the sera of vaccinated cows. VNA also revealed pre-existing antibodies against BVDV in the pre-immunization sera of two cows. To identify BVDV-NS4B specific antibodies, the NS4B protein was expressed in mammalian cells by using the pCI-neo vector system. The sera from BVDV vaccinated cows were evaluated for the presence of BVDV-NS4B specific antibodies through western blot and indirect ELISA. Interestingly, t sera from cows with pre-existing immunity against BVDV were able to detect NS4B in western blot and ELISA, suggesting the presence of NS4B-specific antibodies. The obtained results provide the first indication of the immunogenic nature of BVDV-NS4B protein in sero-converted animals. These findings are consistent with the observation made for NS4B in other *Flaviviridae* members and confirm this protein as an interesting target with diagnostic, vaccination and therapeutic value.

## 1. Introduction

Bovine viral diarrhea virus (BVDV), a pestivirus belonging to the *Flaviviridae* family, is a significant endemic cattle pathogen inflicting substantial economic losses to the cattle industry worldwide. These economic losses are dependent on the clinical manifestation of the disease with acute infections being the major contributor to this squandering. At the cattle population level, overall economic losses account for USD 10–40 million per million of calvings which include production losses, prevention and treatment expenditures [1]. BVDV causes a complex disease affecting different body systems including the respiratory, digestive, reproductive and immune system. It leads to coughing, nasal discharge, diarrhea, immunosuppression and abortion or birth of a persistently infected (PI) calf. These PI animals are immunotolerant to BVDV and shed viruses during their entire life span and serve as a threat to the healthy naïve herd in their proximity [2].

The BVDV genome is comprised of ~12.3 kb long positive sense RNA and contains a long open reading frame (ORF) flanked by 5′- and 3′-untranslated regions (UTR). These UTRs do not code for any protein but rather form secondary structures capable of interacting with each other and also with viral and host proteins to ensure smooth regulation of BVDV RNA replication, transcription and translation. The ORF is translated into a long polypeptide of approximately 4000 amino acids residues, which is subsequently cleaved into structural (C, E^rns^, E1, E2, p7) and non-structural proteins (N^pro^, NS2/3, NS4A, NS4B, NS5A and NS5B) by host and viral proteases [3].

The BVDV NS4B protein is a ~38 kDa hydrophobic protein that serves as a scaffold for viral replication complex [4]. In addition, a single point mutation (Y2441C) in NS4B switches the BVDV virus from a cytopathic to a non-cytopathic biotype, indicating its role in viral pathogenesis [5]. A recent in vitro study has also found a role of BVDV NS4B in the induction of autophagosomes in bovine kidney cells [6]. Apart from these studies, a clear knowledge gap exists in assigning function to this highly hydrophobic protein. Furthermore, BVDV NS4B involvement in viral pathogenesis and immunogenicity is also poorly understood.

Within the *Flaviviridae* family, NS4B homologues in hepatitis C virus (HCV) and dengue virus (DENV) share conserved properties including their membrane topology, genomic location and their role in formatting membranous webs for viral replication [7,8]. In HCV and DENV, NS4B serves as a major immunogenic protein not only in chronic but also in acute infections [9,10]. Recently, NS4B has emerged as major diagnostic [11,12] and therapeutic target [7,8] for HCV and DENV, countering viral infections due to the presence of antigenic epitopes of diagnostic significance and strong immunogenicity.

In BVDV, the principle immunogenicity is ascribed to E^rns^, E2 (structural proteins) and NS3 (non-structural protein). The immunogenic profile of these three proteins is well established and characterized with only the E2 protein capable of eliciting high titers of BVDV-neutralizing antibodies post infection or vaccination [13,14]. Among other BVDV non-structural proteins, the roles of N^Pro^ and NS2 are also well-elaborated [15,16]. Therefore, it is of utmost value to understand and characterize the immunogenic profile of BVDV NS4B in the context of devising better tools to control BVDV infections.

In this study, we investigated the immunogenicity of BVDV NS4B during vaccination with live attenuated BVDV vaccine Bovela^®^. Bovela^®^ is a double deleted BVDV vaccine capable of inducing a strong humoral and T-cell mediated immune response in cattle [17]. To assess the antibody response directed against the BVDV NS4B protein, we performed a virus neutralization test, an indirect ELISA and a western blot.

## 2. Materials and Methods

### 2.1. Animal and Ethical Statement

The vaccination of five cows of the Fleckvieh breed was performed twice, 21 days apart, using the commercial Bovela^®^ vaccine at TRANSfarm facility of KU Leuven, after the approval from the University’s ethical committee (P163/2018). Cows were inducted in the experiment randomly from the cattle lot at TRANSfarm. During the experiment, all the cows had *ad libitum* access to the food and water.

### 2.2. Samples

**Figure 1:**
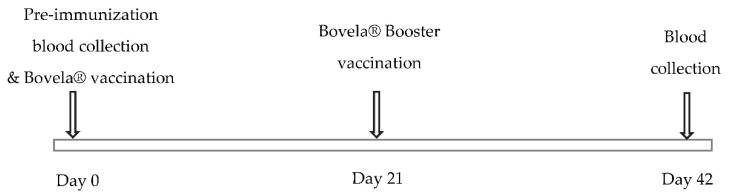
Experimental timeline for the study. Pre-immunization blood collection and Bovela^®^ vaccination was performed on day 0 with booster vaccination on day 21. The second blood collection was performed on day 42 which served as a source of post-immunization sera.

Blood was collected from all cows before (Pre-immunization) and 42 days post first vaccination for serum isolation. All serum samples were kept at −20 °C after isolation. Figure 1 explains the experimental timeline comprising of blood collection and vaccination.

### 2.3. Cells and Viruses

For the viral neutralization assay, BVDV strain NADL was propagated in Madin Darby bovine Kidney (MDBK) cells, cultured in Dulbecco’s modified eagle medium supplemented with 2% fetal bovine serum (FBS), 2% sodium bicarbonate, 1x minimal non-essential amino acids and 1x antibiotic/antimycotic solution. As a positive control for western blot and ELISA analysis, The BVDV-NADL strain was propagated in MDBK cells, cultured in Dulbecco’s modified eagle medium supplemented with 2% horse serum, 2% sodium bicarbonate, 1x minimal non-essential amino acids and 1x antibiotic/antimycotic solution and lysate was prepared. Chinese hamster ovarian (CHO) cells were grown in Ham-F12 medium supplemented with 10% FBS, 2% sodium bicarbonate, 1x minimal non-essential amino acids and 1x antibiotic/antimycotic solution.

### 2.4. NS4B Expression in Mammalian Cells

The pCI-neo-NS4B plasmid was constructed as described in the Supplementary Materials. The pCI-neo-NS4B plasmid was transfected in CHO cells through FuGENE HD transfection reagent (Promega, Leiden, Netherlands) following manufacturer’s instructions. NS4B was purified 72 h post-transfection using Mem-PER™ Plus membrane protein extraction kit (Thermo Fischer Scientific, Brussles, Belgium). The protein concentration of the membrane fraction was quantified with the help of the Pierce BCA protein assay kit (Thermofischer).

### 2.5. Virus Neutralization Assay (VNA)

In all bovine sera employed in this assay, the presence of virus neutralizing antibodies against BVDV-NADL was tested by a standard microtitration procedure as described earlier, but with minor modifications [18]. Briefly, 100 μL of two-fold serially diluted heat inactivated cow sera were mixed with 50 μL of 100 TCID_50_ of BVDV-NADL. This viral-antibody complex was incubated @ 37 °C for 1 h in a humidified CO_2_ incubator. After the incubation, this complex was transferred to MDBK cells seeded as monolayer in 96 well microtiter plate. This whole mixture was incubated for 72 h at 37 °C in a humidified 5% CO_2_ incubator. After this final incubation, an assessment of the virus-induced cytopathic effect was performed microscopically by comparative visual scoring against the negative control. The neutralization titer was defined as the reciprocal of the highest dilution factor of serum capable of fully neutralizing the virus completely and resulting in the absence of viral induced cytopathic effect.

### 2.6. SDS-PAGE and Western Blot

Protein samples were resolved on a 12% SDS-PAGE gel and transferred to nitrocellulose membranes in a semi-dry electrophoretic transfer apparatus (BioRad, Temse, Belgium) using transfer buffer (3.03 g Tris-base, 14.41 g Glycine and 200 mL Methanol in final volume of 1 L) at 100 V for 1 h. Following the protein transfer onto membranes, the blocking was performed overnight at 4 °C in buffer containing 5 mM Tris HCL pH 8, 15 mM NaCl, 0.05% Tween-20 (TBS-T) and 5% bovine serum albumin (BSA). After blocking, washing was performed with TBS-T. Next, the membranes were incubated with cattle serum 1:200 as primary antibody for 1 h in 5% BSA in TBS-T. After three wash cycles with TBS-T, membranes were incubated with 1:6000 HRP conjugated rabbit anti cow antibody (P0159, DAKO, Heverlee, Belgium) for 1 h with 0.1% BSA in TBS-T. Following incubation, membranes were washed three times with TBS-T. The proteins were visualized using an ECL chemiluminescent substrate reagent kit (Thermo Fischer Scientific, Brussels, Belgium) according to manufacturer’s instructions.

### 2.7. Indirect ELISA

To check the efficacy of bovine sera to recognize the NS4B protein, an indirect ELISA was performed as described elsewhere [19]. In short, each well of microtiter plate was coated overnight at 4 °C with 300 ng/well with the membrane protein fractions containing NS4B in 0.1 M carbonate buffer pH 9.6. Lysate of MDBK-BVDV-NADL was used as positive control. After each step, washing was performed five times with washing buffer (PBS, 0.05% Tween-20). Blocking was performed at 37 °C for 1 h with PBS, 0.05% Tween-20, 5% horse serum (PBS-T-HS), 5% skimmed milk powder. Following the addition of 100 μL of diluted bovine serum at 1:200 in PBS-T-HS in each well, incubation was performed at 37 °C for 1 h. Next, 100 μL of 1:5000 HRP conjugated rabbit anti cow antibody (P0159, DAKO, Heverlee, Belgium) in PBS-T-HS was added was applied for 1 h at 37 °C. Finally, ABTS substrate (Thermos Fischer Scientific, Brussels, Belgium) solution was added and absorbance was measured at 405 nm after incubation for 20 min at room temperature.

### 2.8. Statistical Analysis

Data from indirect ELISA were analyzed by drawing box plot by using Microsoft Excel 2016.

## 3. Results

### 3.1. BVDV Vaccination Elicits a Potent Post-Immunization Antibody Response

To evaluate the elicitation of viral neutralizing antibody titer in vaccinated cows, VNA analysis was performed. For this, blood was collected from all cows before (pre-immunization) and 42 days after the first vaccination for serum isolation. These sera were subjected to this assay. Table 1 represents the neutralization titer of pre and post immunization cow sera, respectively. In the pre-immunization sera, cows 1, 2 and 4 did not show any antibody response to the BVDV. The antibody response was evident in the post-immunization sera of the all the cows with the presence of a high neutralizing antibody titer, indicating high magnitude of functional systemic antibodies. Surprisingly, pre-immunized sera of cow 3 and 5 did already show viral neutralizing effect, with high titers of BVDV neutralizing antibodies even at very high dilutions dilution 1:10,240 and 1:5120, respectively (Table 1). A plausible explanation of this sero-conversion could be a prior exposure of these cows to wild type BVDV.

### 3.2. Antigenic Evaluation of NS4B Using Western Blot and ELISA

**Figure 2:**
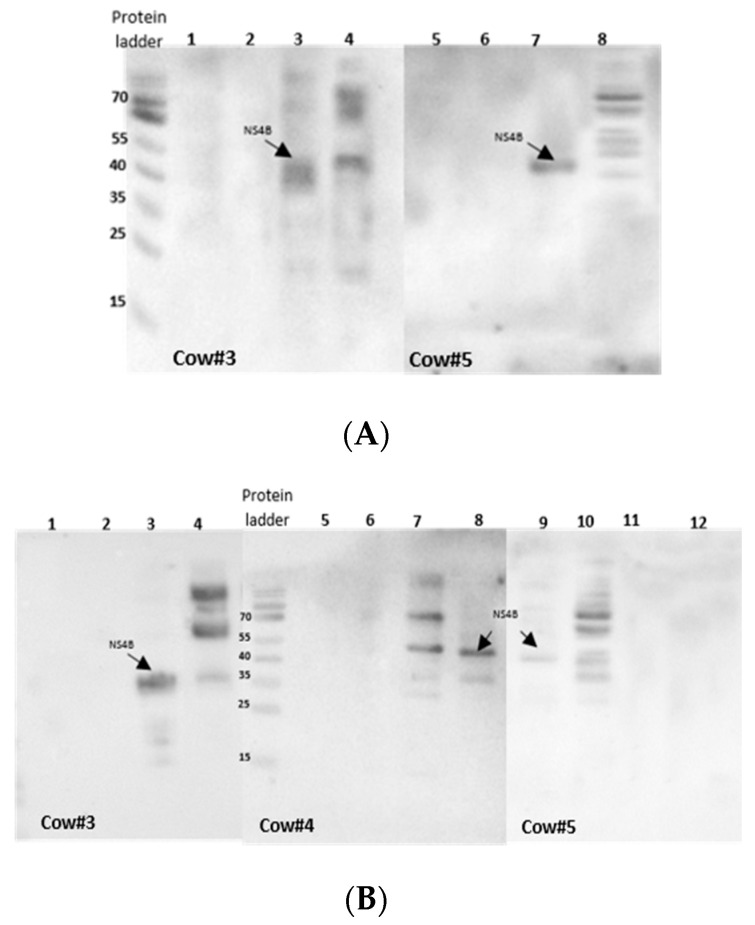
Western blot analysis for detection of NS4B protein with serum from Bovine viral diarrhea virus (BVDV)-vaccinated cows. NS4B was expressed in CHO cells, as outlined in Supplementary Figure S1. After 72 h of transfection, membrane protein fractions were retrieved by the Mem-PER™ Plus kit and separated on 12% SDS-PAGE and transferred to nitrocellulose membrane. (**A**) Western blot with pre-immune sera; NS4B recognized by pre-immune sera of cow 3 and 5. Lane 1 and 5) CHO-empty pCI-neo vector lysate, Lane 2 and 6) Madin Darby bovine Kidney (MDBK) cell lysate, Lane 3 and 7) 38 kDa NS4B recognized by the sera of the cow 3 and 5, respectively, Lane 4 and 8) NADL infected MDBK cell lysate. (**B**) western blot with post-immune sera; Lane 1, 5 and 12) CHO-empty pCI-neo vector lysate, Lane 2, 6 and 11) MDBK cell lysate, Lane 3, 8 and 9) 38kD NS4B protein recognized by the sera of the cow 3, 4 and 5, respectively, Lane 4, 7 and 10) NADL-infected MDBK cell lysate.

**Figure 3:**
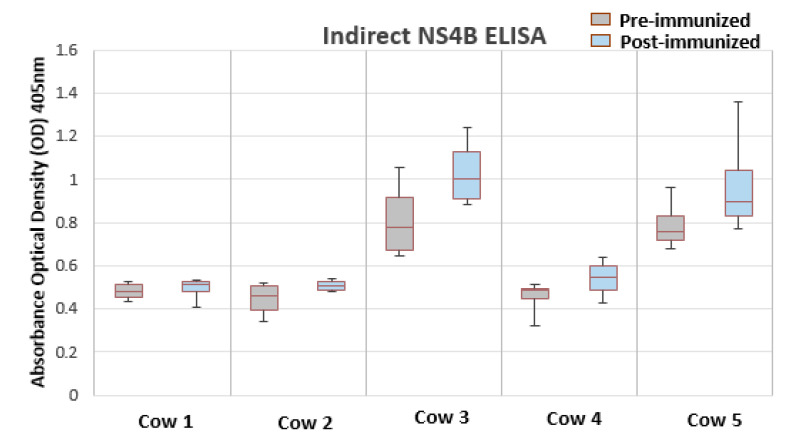
Indirect ELISA for detection of NS4B by using BVDV vaccinated cow sera. Results are being expressed as the difference between the OD405nM obtained with the NS4B and with negative control. Pre- and post-immune sera of cow 3 and 5 are positive for detection of NS4B.

To characterize the antigenicity of BVDV-NS4B protein, western blot and indirect ELISA were performed. For this, BVDV NS4B was produced in CHO cells, as summarized in Figure 2. The antigenicity of BVDV-NS4B was evaluated by using cattle pre-immune and post-immune sera. The 38 kDa NS4B protein could be recognized clearly by both pre- and post-immune sera of cow 3 and 5 as well as post immune sera of cow 4 (Figure 2). A similar result was observed by means of an indirect ELISA where pre-and post-immune sera of the cow 3 and 5 showed higher reactivity toward NS4B but not in case of cow 4 (Figure 3). Pre- and post-immune sera from cow 1 and cow 2 failed to detect NS4B in western blot and ELISA analysis ascertaining the point that sera lacked the antibodies against NS4B protein. The result of all the assays is summarized in the Table 2. Clearly, pre- and post-immune sera of cow 3 and 5 contain viral neutralizing antibodies as evident through VNA and these sera are able to recognize the NS4B protein in western blot and ELISA.

## 4. Discussion

In this study, we investigated the immunogenic role of NS4B in cattle, vaccinated with a modified live Bovela^®^ vaccine by observing the antibody response in sera of these cattle against mammalian cell-expressed NS4B proteins. In terms of characterizing the immunogenic role of the non-structural BVDV-NS4B protein, this is the first study in which the antibody response to this non-structural protein is being reported. For this, polysera of the sero-converted animals were used. This exploratory study is the first step towards unraveling the role of BVDV-NS4B protein role in disease pathogenesis. To assess the vaccine-associated humoral response, VNA was performed. From a biological standpoint, VNA is still considered the a gold standard test to detect and measure the level of neutralizing antibodies for many viral infections including BVDV [19,20]. The results of the VNA elucidated here are measured on MDBK cell culture in a microtiter plate format with constant amount of virus and serially diluted serum samples to an end point where neutralization caused by the virus is no longer detected. VNA revealed the presence of protective antibodies against BVDV in the sera of all post-vaccinated cows, indicating the presence of functional systemic antibodies. Interestingly, two cows already showed a high neutralizing antibody titer even in the pre-immune sera, hinting at a pre-existing immunity attributed to the exposure of these animals to the wild-type BVDV strain.

Western blot and indirect ELISA were performed to unravel the immunogenicity of NS4B by exposing it the pre- and post-immune sera of cows. Pre- and post-immune sera of cow 1 and 2 failed to recognize BVDV-NS4B proteins in western blot and ELISA, even though their post-immune sera showed high viral neutralization titer. The ability of the of pre- and post-immune sera of the cow 3 and 5, which already showed presence of neutralizing antibodies in VNA, in recognizing the NS4B in western blot and indirect ELISA may reflect the necessity of repeated infections and a longer exposure time to induce NS4B specific antibodies.

As stated previously, the presence of pre-existing immunity in two of these cows might be due to previous exposure to the BVDV wild-type strain. Such wild-type strains have unlimited replication potential, and this might have elicited a more robust antibody response in general and also NS4B-specific response. On the other hand, Bovela is a double deletion mutant vaccine derived from the highly pathogenic BVDV strain. This attenuation renders the virus incapable of crossing the placenta thus limiting its replication potential *in vivo*. This might also be the reason for the failure to elicit NS4B-specific antibodies. Although post-immune sera of one cow recognized the NS4B in western blot analysis, in spite of the lack of a neutralizing antibody titer in the pre-immune sera, bit failed to be detected in ELISA. One possible reason for this discrepancy could be the failure of correct presentation of antigenic epitope ELISA to serum antibodies

From this exploratory study, it is evident that BVDV-NS4B shares this inherited characteristic similarity in immunogenicity with HCV-NS4B [9] as compared to that of other members of the Flaviviridae family. In the case of the Zika virus and Dengue virus, consistent serum antibody response has been observed against NS4B even in acute infections [10,21]. In these viruses, the immunogenic role of NS4B and other non-structural proteins has been well-established using the same methodology as adopted in this study [9,10,15]. The role of NS4B as a potential antiviral therapeutic target and vaccine candidate has already been well established in other members of the *Flavivirida* family [22,23,24,25,26,27] and could be actively pursued to control viral infections belonging to BVDV. Future exploration of anti-NS4B antibodies in acute and chronic infections and epitope mapping could contribute further to our current understanding of NS4B’s immunogenicity during BVDV pathogenesis and its assessment as a vaccine candidate.

Investigations into viral non-structural proteins could yield more accurate information about their potential role in immunogenicity and lead to the development of a diagnostic tool and further unravelling of the immunopathology of the disease in different types of infection and clinical manifestations. In this regard, several non-structural proteins have been successfully exploited to evaluate antibody responses to viral infections belonging to family Flaviviridae and led to the differentiation of different diseases stages and clinical manifestations [10,28,29,30,31]. Since NS4B plays a significant role in eliciting humoral and cellular immunity in other viral infections, initial commercial diagnostic assays were based on this protein due to harboring of highly conserved epitopes of diagnostic significance [32,33,34]. In case of BVDV, diagnosis and control still largely remain onerous owing to its various clinical manifestations such as acute, chronic, persistent infection and mucosal disease. This requires a continuous development of robust diagnostic tools which could fulfill the needs of varying situations and infections. In this context, BVDV-NS4B could serve as a useful tool not only in establishing a commercial diagnostic assay but also in differential diagnostics of infected and vaccinated animals. Further studies need to be conducted to delineate the epitopes of this non-structural protein responsible for humoral and cellular immune responses.

## 5. Conclusions and Perspectives

To the best of our knowledge, this is the first study to explore the immunogenic property of BVDV-NS4B in BVDV-vaccinated cows, where polysera of sero-converted cows successfully detected this non-structural protein. This could lead towards better understanding of this non-structural protein and its role in diagnosis, prognosis and immunopathology of the BVDV infection. This will reveal new insights in the immunogenicity of the BVDV-NS4B protein in viral pathogenesis and lead to the establishment of better diagnostic tools in the BVDV control program. In future, epitope mapping and the presence of anti-NS4B antibodies in acute and chronic infections could lead to the development of better diagnostic tools.

## Figures and Tables

**Table 1:** Neutralization titers of the serum samples reactive in virus neutralization assay.

Cow Number	Pre-Immune Neutralization Titre Dilutions *	Post-Immune Neutralization Titre Dilutions *
1	-	1:10,240
2	-	1:10,240
3	1:10,240	1:10,240
4	-	1:2560
5	1:5120	1:5120

* Highest dilution of the serum capable of protecting the cells from virus induced cytopathic effect.

**Table 2:** Results (+/−) are denoted for different tests: viral neutralization assay, indirect ELISA based on NS4B and western blot against BVDV NS4B.

Animal	Status	VNA	W.B	ELISA
Cow 1	Pre-Immunization	−	−	−
	Post-immunization	+	−	−
Cow 2	Pre-Immunization	−	−	−
	Post-immunization	+	−	−
Cow3	Pre-Immunization	+	+	+
	Post-immunization	+	+	+
Cow4	Pre-Immunization	−	−	−
	Post-immunization	+	+	−
Cow5	Pre-Immunization	+	+	+
	Post-immunization	+	+	+

VNA: Virus neutralization assay; W.B.: Western blot; ELISA: Enzyme-linked immunosorbent assay; + = Positive; − = Negative.

## Supplementary Materials

The following are available online at https://www.mdpi.com/2306-7381/7/4/169/s1, Figure S1: Cloning strategy of BVDV-NS4B gblock in pCI-neo vector. A) pCI-neo-NS4B vector map, where NS4B is cloned with restriction sites XhoI and XbaI. NS4B expression is controlled by CMV promoter. B) Colony PCR after ligation of cut BVDV-NS4B g block and cut pCI-neo vector and transformation of ligation mix and resolution on 1.5% agarose gel; Lane 1: 100 base pair ladder, Lane 2: negative control, Lane 3: Colony PCR mix with NS4B DNA band of 1100 base pair.

## References

[1] Houe H. (2003). Economic impact of BVDV infection in dairies. Biologicals.

[2] Lanyon S.R., Hill F.I., Reichel M.P., Brownlie J. (2014). Bovine viral diarrhoea: Pathogenesis and diagnosis. Vet. J..

[3] Neill J.D. (2013). Molecular biology of bovine viral diarrhea virus. Biologicals.

[4] Weiskircher E., Aligo J., Ning G., Konan K.V. (2009). Bovine viral diarrhea virus NS4B protein is an integral membrane protein associated with Golgi markers and rearranged host membranes. Virol. J..

[5] Qu L., McMullan L.K., Rice C.M. (2001). Isolation and Characterization of Noncytopathic Pestivirus Mutants Reveals a Role for Nonstructural Protein NS4B in Viral Cytopathogenicity. J. Virol..

[6] Suda Y., Murakami S., Horimoto T. (2018). Bovine viral diarrhea virus non-structural protein NS4B induces autophagosomes in bovine kidney cells. Arch. Virol..

[7] Wang Z., Chen X., Wu C., Xu H., Liu H. (2016). Current Drug Discovery for Anti-hepatitis C Virus Targeting NS4B. Curr. Top. Med. Chem..

[8] Xie X., Zou J., Wang Q.-Y., Shi P.-Y. (2015). Targeting dengue virus NS4B protein for drug discovery. Antivir. Res..

[9] Sillanpää M., Melén K., Porkka P., Fagerlund R., Nevalainen K., Lappalainen M., Julkunen I. (2009). Hepatitis C virus core, NS3, NS4B and NS5A are the major immunogenic proteins in humoral immunity in chronic HCV infection. Virol. J..

[10] Lazaro-Olán L., Mellado-Sánchez G., García-Cordero J., Escobar-Gutiérrez A., Santos-Argumedo L., Gutiérrez-Castañeda B., Cedillo-Barrón L. (2008). Analysis of Antibody Response in Human Dengue Patients from the Mexican Coast Using Recombinant Antigens. Vector Borne Zoonotic Dis..

[11] Amin N., Pupo M., Aguilar A., Vázquez S., Caballero Y., Ochoa R., Guzman M.G., Acosta A. (2016). Recognition of a multiple antigen peptide containing sequence from mimotope of the dengue type 3 virus NS4B protein by human antibodies. Asian Pac. J. Trop. Med..

[12] Savardashtaki A., Sharifi Z., Hamzehlou S., Farajollahi M.M. (2015). Analysis of Immumoreactivity of Heterologously Expressed Non-structural Protein 4B (NS4B) from Hepatitis C Virus (HCV) Genotype 1a. Iran. J. Biotechnol..

[13] Donis R.O., Corapi W., Dubovi E.J. (1988). Neutralizing Monoclonal Antibodies to Bovine Viral Diarrhoea Virus Bind to the 56K to 58K Glycoprotein. J. Gen. Virol..

[14] Bolin S.R. (1993). Immunogens of bovine viral diarrhea virus. Vet. Microbiol..

[15] Mishra N., Rajukumar K., Pitale S.S., Prakash A., Nema R.K., Behera S.P., Dubey S.C. (2010). Evidence of a humoral immune response against the prokaryotic expressed N-terminal autoprotease (Npro) protein of bovine viral diarrhoea virus. J. Biosci..

[16] Agapov E.V., Murray C.L., Frolov I., Qu L., Myers T.M., Rice C.M. (2004). Uncleaved NS2-3 Is Required for Production of Infectious Bovine Viral Diarrhea Virus. J. Virol..

[17] Platt R., Kesl L., Guidarini C., Wang C., Roth J.A. (2017). Comparison of humoral and T-cell-mediated immune responses to a single dose of Bovela ® live double deleted BVDV vaccine or to a field BVDV strain. Veter- Immunol. Immunopathol..

[18] Gauger P.C., Vincent A.L. (2014). Serum Virus Neutralization Assay for Detection and Quantitation of Serum-Neutralizing Antibodies to Influenza A Virus in Swine. Recent Results Cancer Res..

[19] Zoth S.C., Taboga O. (2006). Multiple recombinant ELISA for the detection of bovine viral diarrhoea virus antibodies in cattle sera. J. Virol. Methods.

[20] Newman F.K., Frey S.E., Blevins T.P., Mandava M., Bonifacio J.A., Yan L., Belshe R.B. (2003). Improved Assay to Detect Neutralizing Antibody following Vaccination with Diluted or Undiluted Vaccinia (Dryvax) Vaccine. J. Clin. Microbiol..

[21] Li G., Adam A., Luo H., Shan C., Cao Z., Fontes-Garfias C.R., Sarathy V.V., Teleki C., Winkelmann E.R., Liang Y. (2019). An attenuated Zika virus NS4B protein mutant is a potent inducer of antiviral immune responses. NPJ Vaccines.

[22] Van Cleef K.W.R., Overheul G.J., Thomassen M.C., Marjakangas J.M., Van Rij R.P. (2016). Escape Mutations in NS4B Render Dengue Virus Insensitive to the Antiviral Activity of the Paracetamol Metabolite AM404. Antimicrob. Agents Chemother..

[23] Pouliot J.J., Thomson M., Xie M., Horton J., Johnson J., Krull D., Mathis A., Morikawa Y., Parks D., Peterson R. (2015). Preclinical Characterization andIn VivoEfficacy of GSK8853, a Small-Molecule Inhibitor of the Hepatitis C Virus NS4B Protein. Antimicrob. Agents Chemother..

[24] Xu J., Xie X., Ye N., Zou J., Chen H., White M.A., Shi P.-Y., Zhou Z. (2019). Design, Synthesis, and Biological Evaluation of Substituted 4,6-Dihydrospiro[[1,2,3]triazolo[4,5- b]pyridine-7,3′-indoline]-2′,5(3 H)-dione Analogues as Potent NS4B Inhibitors for the Treatment of Dengue Virus Infection. J. Med. Chem..

[25] Roth C., Cantaert T., Colas C., Prot M., Casadémont I., Levillayer L., Thalmensi J., Langlade-Demoyen P., Gerke C., Bahl K. (2019). A Modified mRNA Vaccine Targeting Immunodominant NS Epitopes Protects Against Dengue Virus Infection in HLA Class I Transgenic Mice. Front. Immunol..

[26] Kuhs K.A.L., Toporovski R., Ginsberg A.A., Shedlock D.J., Weiner D.B. (2012). Induction of Intrahepatic HCV NS4B, NS5A and NS5B-Specific Cellular Immune Responses following Peripheral Immunization. PLoS ONE.

[27] Latimer B., Toporovski R., Yan J., Pankhong P., Morrow M.P., Khan A.S., Sardesai N.Y., Welles S.L., Jacobson J.M., Weiner D.B. (2014). Strong HCV NS3/4a, NS4b, NS5a, NS5b-specific cellular immune responses induced in Rhesus macaques by a novel HCV genotype 1a/1b consensus DNA vaccine. Hum. Vaccines Immunother..

[28] Berasain C., García-Granero M., Riezu-Boj J.I., Civeira M.P., Banales J.M., Borrás-Cuesta F. (1993). Detection of anti-hepatitis C virus antibodies by ELISA using synthetic peptides. J. Hepatol..

[29] Valdés K., Alvarez M., Pupo M., Vázquez S., Rodríguez R., Guzmán M.G. (2000). Human Dengue Antibodies against Structural and Nonstructural Proteins. Clin. Diagn. Lab. Immunol..

[30] Kuno G., Vorndam A.V., Gubler D.J., Gómez I. (1990). Study of anti-dengue NS1 antibody by Western blot. J. Med. Virol..

[31] Wong S.J., Boyle R.H., Demarest V.L., Woodmansee A.N., Kramer L.D., Li H., Drebot M., Koski R.A., Fikrig E., Martin D.A. (2003). Immunoassay Targeting Nonstructural Protein 5 To Differentiate West Nile Virus Infection from Dengue and St. Louis Encephalitis Virus Infections and from Flavivirus Vaccination. J. Clin. Microbiol..

[32] Conrycantilena C. (1997). Hepatitis C virus diagnostics: Technology, clinical applications and impacts. Trends Biotechnol..

[33] Masalova O.V., Lakina E., Abdulmedzhidova A., Atanadze S., Semiletov Y., Shkurko T., Burkov A., Ulanova T., Pimenov V., Novikov V. (2002). Characterization of monoclonal antibodies and epitope mapping of the NS4 protein of hepatitis C virus. Immunol. Lett..

[34] Chang J., Seidel C., Ofenloch B., Jue D., Fields H., Khudyakov Y. (1999). Antigenic Heterogeneity of the Hepatitis C Virus NS4 Protein as Modeled with Synthetic Peptides. Virology.

