# Supplementary material for: Deciphering the Role of Bovine Viral Diarrhea Virus Non-Structural NS4B Protein in Viral Pathogenesis
Source: Vet Sci. 2020 Oct 31;7(4):169. doi: 10.3390/vetsci7040169 (PMC7712251; doi:10.3390/vetsci7040169)
Supplement: Supplementary file 1 [file vetsci-07-00169-s001.pdf]

veterinary sciences

MDPI

Supplementary Data:

BVDV-NS4B Gblock Cloning in pCI-Neo Vector

NS4B (GenBank ID: KX170538.1) gblock was ordered from IDT technologies (Haasrode, Belgium). Restriction digestion of NS4B gblock and pCI-neo vector (Promega, Cat# E1841) was performed by using XhoI and XbaI restriction enzymes. Following restriction, NS4B gblock and pCI-neo vector were ligated using T4 DNA ligase (Promega). The ligation mix was transformed in electro-competent XL1-Blue E.coli through electroporation and plated onstandard lysogeny broth (LB)/Ampicillin agar plates for overnight incubation at 37 °C. Single colony was picked and grown overnight in LB medium for PureYield plasmid miniprep (Promega). Cloning was confirmed by colony PCR and sequencing using plasmid specific primers (T7 EEV: 5′-AAGGCTAGAGTACTTAATACGA-3′; T3: 5′-ATTAACCCTCACTAAAGGGA-3′). After confirmation, the pCI-neo-NS4B cloned plasmid was transfected to CHO cells through FuGENE HD transfection reagent (Promega) by following manufacturer's instructions. NS4B was purified 48 hours post-transfection using Mem-PER™ Plus membrane protein extraction kit (Thermofischer). Concentration of membrane fraction proteins was quantified with the help of Pierce BCA protein assay kit (Thermofischer).

Using restriction cloning, we successfully cloned BVDV-NS4B gblock into pCI-neo vector (Figure S1 (A)). Figure S1 (B) represents the colony PCR with NS4B at the height of 1100 bp. Cloning was also confirmed by sequencing the purified plasmid pCI-neo-NS4B.

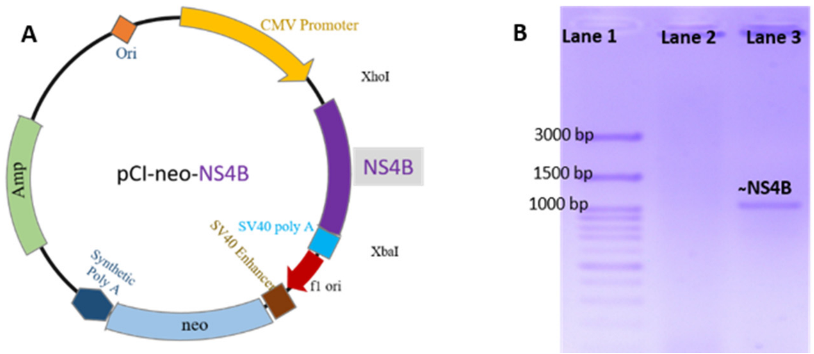

Figure S1. Cloning strategy of BVDV-NS4B gblock in pCI-neo vector. A) pCI-neo-NS4B vector map, where NS4B is cloned with restriction sites XhoI and XbaI. NS4B expression is controlled by CMV promoter. B) Colony PCR after ligation of cut BVDV-NS4B g block and cut pCI-neo vector and transformation of ligation mix and resolution on 1.5% agarose gel; Lane 1: 100 base pair ladder, Lane 2: negative control, Lane 3: Colony PCR mix with NS4B DNA band of 1100 base pair.
